# The first nosocomial outbreak of methicillin-resistant *Staphylococcus aureus *in horses in Sweden

**DOI:** 10.1186/1751-0147-54-11

**Published:** 2012-02-08

**Authors:** Karin Bergström, Anna Aspan, Annica Landén, Christopher Johnston, Ulrika Grönlund-Andersson

**Affiliations:** 1 Faculty of Veterinary Medicine and Animal Husbandry, Swedish University of Agricultural Sciences, 750 07 Uppsala, Sweden; 2 Department of Bacteriology, SVA, 751 89 Uppsala, Sweden; 3 Department of Animal Health and Antimicrobial Strategies, SVA, 750 89 Uppsala, Sweden; 4 Equine Clinics, University Animal Hospital, University of Agricultural Sciences, 750 07 Uppsala, Sweden

**Keywords:** Methicillin-resistant, *Staphylococcus aureus*, MRSA, horses, outbreak, pulsed-field gel electrophoresis, PFGE

## Abstract

**Background:**

Methicillin-resistant *Staphylococcus aureus *(MRSA) in animals is a rare finding in Sweden. In horses, MRSA was first detected in a screening survey in 2007. In 2008, six clinical cases occurred in an equine hospital, indicating an outbreak.

**Method:**

All MRSA isolates detected, 11 *spa*-type t011 and one t064 (n = 12), in infected horses (n = 10) and screening of horses (n = 2) in Sweden from December 2007 to March 2010 were retrospectively analysed with pulsed-field gel electrophoresis (PFGE) using *Cfr*9I and *Apa*I restriction enzymes, to study relationship between the isolates. Medical records of infected horses and outbreak investigation notes were scrutinised to monitor the clinical outcome and other aspects of the outbreak.

**Results:**

Eight of the 10 infected horses were linked to one equine hospital and two to another hospital in the same region. The six horses infected with MRSA in 2008 underwent surgery during the period 22 May-7 July in one of the hospitals. Four more infections linked to the two hospitals were notified between 2009 and March 2010.

Nine of the 11 *spa*-type t011 isolates had identical *Cfr*9I and *Apa*I PFGE pattern. All six infected horses from 2008 presented with this MRSA. Two t011 isolates differed in one and two bands, respectively, in PFGE.

Nine horses suffered from surgical site infections (SSI). No antimicrobials were used following the MRSA diagnosis and the infections cleared. The time from surgery to MRSA diagnosis differed greatly between the horses (range 15-52 days).

**Conclusions:**

Association in time and space of six horses infected with an identical MRSA strain of *spa*-type t011 confirmed an outbreak. Two isolates found in 2009 and 2010 in the outbreak hospital were closely related to the outbreak strain, indicating one circulating strain. Both *spa*-type t011 and t064 have been reported in horses in Europe prior to these findings. The observation that the infections cleared although antimicrobials were not used is encouraging for future prudent use of antimicrobials. The time from surgery to bacteriological diagnosis was not acceptable in most cases, as contagious spread was a risk. Sampling when symptoms of infection are noticed and accurate analysis are thus important.

## Background

*Staphylococcus (S.) aureus *is a potentially pathogenic bacterium causing pyogenic infections in both man and animals. Nosocomial and community-acquired infections with therapeutic limitations due to methicillin-resistant *S. aureus *(MRSA) have become a worldwide problem in humans [1-5]. However, according to the European Antimicrobial Resistance Surveillance Network annual report for 2010, a significant decrease in human MRSA rates has occurred in seven European countries in recent years, which might be the result of successful preventive measures [6].

An increasing number of different animal species colonised or infected with MRSA has also been reported during recent decades [7-15]. The first report of MRSA in horses was in brood mares with endometritis in Japan [8,9], but surgical site infections (SSI) and traumatic wound infections are more frequently reported [10,12,14,15]. MRSA associated with livestock, of multi locus sequence type (MLST) ST398, has emerged in Europe [15-19]. People with professions involving contact with animals carrying MRSA ST398 are reported to be colonised with this type to a higher extent than the average population [2,15,20-23]. Within the cluster of ST398 one *spa*-type, t011, is predominant in pigs in Europe and has also been found e.g. in rats on pig farms and in cattle [19,24-27]. *Spa*-type t011 has also been detected in horses and in equine hospital environments [15,17,18,28,29]. Human infections with this *spa*-type have been reported in Europe [30-32]. MRSA of *spa*-type t064, belonging to ST8, has also been described in horses in Europe, although not as frequently as *spa*-type t011 [12,15].

Pulsed-field gel electrophoresis (PFGE) has long been the 'gold standard' for fingerprinting MRSA using *Sma*I endonuclease, but ST398 is not typeable by the standardised method [33]. Other enzymes, such as *Cfr*91, *Apa*I and *Sac*II, have proven useful for cutting DNA in ST398 strains [24,34-37].

MRSA and other methicillin-resistant, coagulase-positive staphylococci detected in animals are notifiable since 1 January 2008, according to Swedish legislation (SJVFS 2007:90). MRSA is rare in animals in Sweden according to: i) official notification data from the Swedish Board of Agriculture (SJV); ii) ongoing annual monitoring of antimicrobial-resistant bacteria since 2000 in the Swedish Veterinary Antimicrobial Resistance Monitoring (SVARM) programme; and iii) targeted screening surveys of pigs and horses within the country [38-40]. However, in 2010 (some months after this study was completed) for the first time a Swedish pig herd, one of 191 tested in a national screening programme, was confirmed as MRSA-positive. The isolate belonged to ST398, *spa*-type t011 [39]. MRSA has so far not been detected in cattle in Sweden [39]. The first documented MRSA isolate of equine origin in Sweden was from a screening survey in 2007, when one of 300 healthy horses presented with ST398, *spa*-type t011, in a nasal sample [38].

The aim of this study was to employ PFGE to further analyse all available MRSA isolates from horses in Sweden, obtained between 2007 and 2010, in order to establish whether a nosocomial outbreak had occurred and whether sporadic cases could be connected to this suspected outbreak. An additional aim was to describe the clinical conditions of the suspected outbreak and the outcome for the horses.

## Methods

### Design of the study

Retrospective analyses were used to investigate the genetic relatedness between 12 MRSA isolates detected between December 2007 and March 2010 in horses in Sweden, in order to establish whether a nosocomial outbreak had occurred. The outcome for the horses and other factors of importance were studied by gathering data from the medical records of infected horses, hospital notes on surgeries and outbreak investigation documents.

### Isolates

The 12 isolates analysed with PFGE were provided by the Department of Animal Health and Antimicrobial Strategies at the Swedish National Veterinary Institute (SVA), which collects isolates of antimicrobial-resistant bacteria and carries out typing and antimicrobial susceptibility testing. The isolates represented all MRSA detected in horses in Sweden in the study period and originated from clinical sampling of wound infections (n = 10) using swabs for routine bacteriological sampling or from nasal screening (n = 2), as listed below and in Table 1.

**Table 1:** Methicillin-resistant *Staphylococcus aureus *in horses in Sweden, December 2007 to March 2010.

Year	Date of surgery	Type of surgery	Inf^1)^	Pulsotype^2)^	Hospital (MRSA case)	Outcome/comments
**2007**	NA **^3)^**	NA, nasal screening	NA	A	NA	First known MRSA case in horses in Sweden [38]
**2008**	May 22	Tenoscopy	SSI	A	1 (Horse I)	Euthanized 2 June
	May 26	Ovariectomy	SSI	A	1 (II)	Healed, sampled twice, neg on 1^st ^occasion, pos on 2^nd^
	Jun 2	Ovariectomy	SSI	A	1 (III)	Healed
	Jul 2	Ovariectomy	SSI	A	1 (IV)	Healed
	Jul 4	Umbilical hernia	SSI	A	1 (V)	Healed, found in phone tracing
	Jul 7	Colic	SSI	A	1 (VI)	Healed, sampled twice, neg on 1^st ^occasion, pos on 2^nd^
	NA	NA, nasal screening	NA	A	NA	NA, contact tracing
**2009**	Aug 25	Extensor tendon rupture	SSI	A2	2 (VII)	Healed
	Aug **^4)^**	NA, inf. pressure wound	TW	A	1 (VIII)	Healed
**2010**	Feb 4	Tenectomy	SSI	A1	1 (IX)	Healed
	Feb 27	Colic	SSI	B	2 (X)	Healed, culture by hospital lab, ST8, spa-type t064

Isolates were of ST 398, *spa*-type t011, unless otherwise stated.

**Figure 1:**
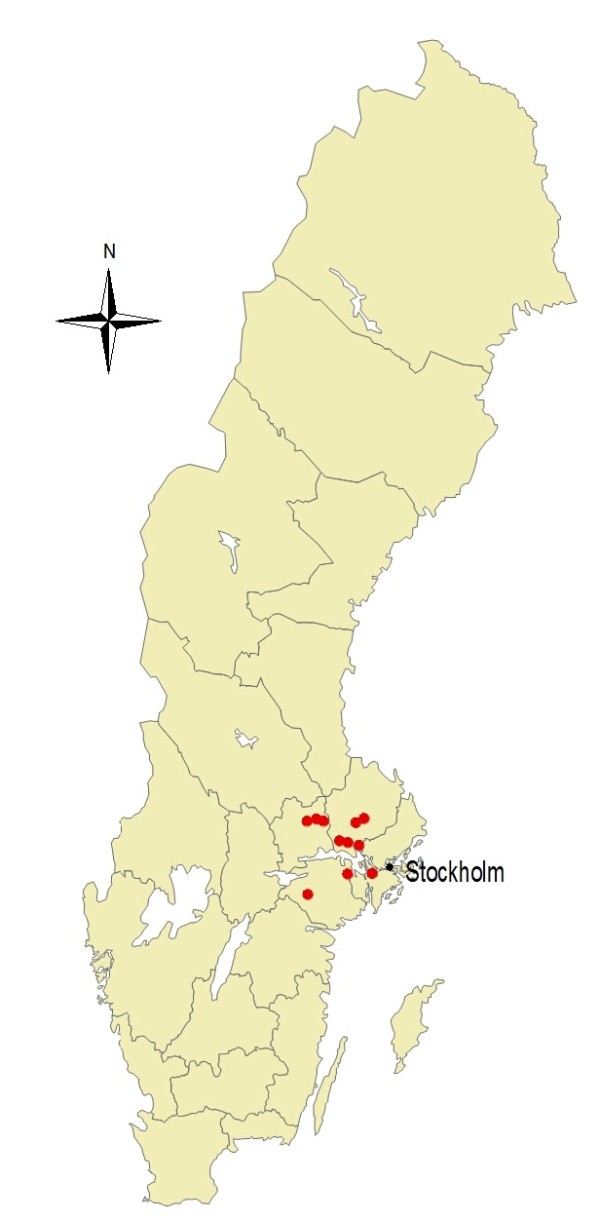
**Red dot indicate approximate location of MRSA-infected horses in Sweden, June 2008 to March 2010**.

1) Type of infection; surgical site infection (SSI), traumatic wound (TW).

2) PFGE pulsotype, *Apa*I and *Cfr*91 restriction enzyme, see Figure 1 for details.

3) NA = not applicable or not known.

4) Foal, admission in June 2009 for neonatal care. In August a second visit, surgery due to a broken injection needle in a muscle which healed. Third visit, a pressure sore in a front leg due to bandage in which MRSA was detected in Sept 2009.

- Eight isolates were linked to one equine hospital (Hospital 1), six infected during the summer of 2008 (Horse I, II, III, IV, V and VI) and two sporadic infections, in 2009 (Horse VIII) and 2010 (Horse IX).

- Two isolates were linked to another hospital in the same region (Hospital 2), and originated from two sporadically infected horses, in 2009 (Horse VII) and 2010 (Horse IX).

- Two isolates came from non-infected horses with positive nasal cultures; one in an anonymous screening survey in 2007, where one of 300 horses tested positive [38]; and one from screening of horses that had been in contact with the infected horses in 2008, where one of 14 horses tested positive.

#### Culture, susceptibility testing, confirmation of methicillin resistance and genotyping of the isolates prior to PFGE

The isolates had been analysed at SVA and the Swedish Institute for Communicable Disease Control (SMI) as described below before being submitted to this retrospective study.

One sample was cultured in an equine hospital's own laboratory and the isolate was sent to SVA for MRSA confirmation and further typing (Table 1). For this isolate, the culture method is not known. The remaining samples were cultured at SVA. Clinical swabs were routinely streaked onto a 5% bovine blood and Bromcresole Purple Lactose agar plate (both SVA), incubated for 24 to 48 hours at 37°C and checked after 24 and 48 hours for bacterial growth. When MRSA was suspected by the sampling clinician, the swab was also streaked onto Brilliance MRSA agar (Oxoid, Basingstoke, UK) and plates were incubated at 37°C for 24 to 48 h.

The two nasal swabs from screening (Table 1) were selectively cultivated for MRSA according to Busscher et al. [41], with the exception that cefoxitin (1 mg/L) was used instead of ceftizoxime. Suspected colonies from the cultivation having typical morphology, such as haemolysin production on blood agar or blue colonies on Brilliance MRSA agar, were recultivated and further phenotyped with biochemical testing (Gram staining, catalase, coagulase and maltose fermentation). Biochemically verified *S. aureus *was further tested as described below. The isolate cultured in the hospital laboratory was also tested in this way.

In addition to demonstrating resistance to betalactams, all isolates provided had proved resistant to ciprofloxacin, gentamicin, kanamycin, tetracycline and trimethoprim and susceptible to erythromycin, clindamycin and fusidic acid in previous antimicrobial susceptibility testing of the isolates. This was performed by broth microdilution according to standards of the Clinical and Laboratory Standards Institute guidelines, document M100-S17 [42], using microdilution panels (VetMIC™, SVA, Uppsala, Sweden). *S. aureus *CCUG 15915 was used as quality control. Resistance was defined using epidemiological cut-off values according to the European Committee on Antimicrobial Susceptibility Testing [43].

All isolates were confirmed as MRSA by polymerase chain reaction (PCR) analyses for the *mecA *gene and the *S. aureus *species-specific *nuc *gene according to Smyth et al. [44]. Eleven of the 12 isolates were of *spa*-type t011 (tandem repeats 8-16-2-25-34-22-25) and one of *spa*-type t064 (tandem repeats 11-19-12-05-17-34-24-34-22-25). *Spa*-typing was performed according to Harmsen et al. [45] by SMI or SVA. Two *spa*-type t011 isolates analysed with MLST according to Enright et al. [46] were identified as ST398 and the t064 as ST8. MLST of these isolates was carried out by SMI to confirm expected sequence type identity, as the *spa*-types were known.

### Horses

Data on the date and type of surgery, antimicrobial treatment, surgical hospital, length of time from the date of surgery to MRSA detection, progress and clinical outcome were collected from medical records on the horses. Post-surgery notes and outbreak investigation notes from Hospital 1 provided information relating to the outbreak.

### Pulsed-field gel elctrophoresis

Macro-restriction analysis of the isolates was performed at SVA. As the restriction enzyme typically used for PFGE of MRSA, *Sma*I, is blocked by methylation of specific recognition sites in ST398 strains [37], the enzymes *Cfr*9I, a neoschizomer of *Sma*I, and *Apa*I were chosen instead [24,34-36] and used according to the HARMONY protocol [33]. Chromosomal DNA of *S. aureus *NCTC 8325 was used as the reference size marker for normalisation of PFGE gels. Banding patterns were compared visually and the level of similarity between patterns used for defining pulsotypes was a minimum of one band difference, according to guidelines devised by Tenover et al. [47].

## Results

### Horses and outbreak data

Where the horses lived was known for 11 of the 12 horses and their home stables were located in a region approximately between 20 and 200 km from the Swedish capital, Stockholm (Figure 1). The screening in 2007 was anonymous and thus no data were available for this horse. Date and type of surgery, type of infection, surgical hospital and outcome for the infected horses are shown in Table 1. No medical records were available for the two non-infected horses that tested positive in the screening studies.

The first MRSA case (Table 1 Horse I) in the outbreak related to Hospital 1 underwent surgery on 22 May and was diagnosed as infected in June 2008. During the summer of 2008 five more horses became infected (Table 1 Horses II, III, IV, V and VI). In tracing for a possible source, it was noted that the three horses that underwent surgery between 22 May and 2 June (Horses I, II and III) had indirect contacts within the hospital. Horse I was readmitted to the hospital four days after surgery because of swelling in the surgery area and the operated tendon sheath was flushed both before and after horse II underwent surgery in the same theatre. During the last flushing of Horse I, on 2 June, a bacteriological sampling was performed and the horse was then euthanized due to guarded prognosis. On 2 June Horse III also underwent surgery (laparoscopy) in another surgical theatre, but with instruments used previously for the tenoscopy on Horse I. Instruments had been routinely cleaned and disinfected or autoclaved between patients. Thus in summary, Horses I and II had indirect contact through the operating theatre and Horses I and III through surgical instruments. Horses I, II and III were all born in Sweden and had no history of being abroad, but contacts with other horses within Sweden and their origin and cross-connections could not be tracked down when searching for possible transmission outside the hospital. The next cluster of three infected horses (Table 1 Horses IV, V and VI) had surgery within a five-day period, 2-7 July, about a month later.

After the outbreak in summer 2008, four more horses infected with MRSA were notified in Sweden by March 2010; two in Hospital 1 and two linked to Hospital 2 (Table 1).

According to medical records, infections were superficial in eight of the 10 infected horses. The infection in the tendon sheath of Horse I was recorded as severe, but as this horse was euthanized the outcome of the infection could not be evaluated. The wound infection of the horse with the ruptured extensor tendon (Horse VII) had visible bone but intact periosteum and was also judged as rather severe. Medical treatment before MRSA diagnosis was not recorded for all horses, but when MRSA was diagnosed no antimicrobials were administered to the nine infected horses still alive (Horse I already euthanized). According to the medical records, the infections cleared and wounds healed in all the horses evaluated.

The entire time span from the day of surgery to laboratory confirmation of MRSA differed widely between the horses, ranging from 15 to 52 days (median 26 days). Five of the 10 infected horses were sampled in the hospital, one still hospitalised after surgery (Horse VI) and four when readmitted because of infected non-healing wounds (Horses I, VIII, IX and X). The remaining five were sampled at home and of those was one traced by telephone (Horse V) as described below (Table 1). Of the six outbreak horses two were sampled twice as their infections did not clear and MRSA was not detected on the first but on the second sampling occasion (Horses II and VI). The remaining infected horses tested positive for MRSA on the first sampling occasion. The history of antimicrobial treatment before sampling could not be determined for all horses, but at least one horse (IX) was treated with penicillin for a week, without effect, before sampling. One of the horses (Horse V) in the outbreak was found in a telephone tracing of owners of horses that underwent surgery within the outbreak period, 22 May-7 July, when searching for potentially infected horses connected to Hospital 1. Of 45 listed owners 37 were reached, four reported clinical signs of infection in their horses, three horses were sampled and one tested positive. The time from bacteriological sampling of horses with infections to laboratory confirmation of MRSA ranged between three and 10 days (median 7 days). The time taken for analysis was influenced by variable quality of samples, laboratory closure on weekends and parcel delivery time.

There were no known direct connections between Hospitals 1 and 2.

### Genotyping

**Figure 2:**
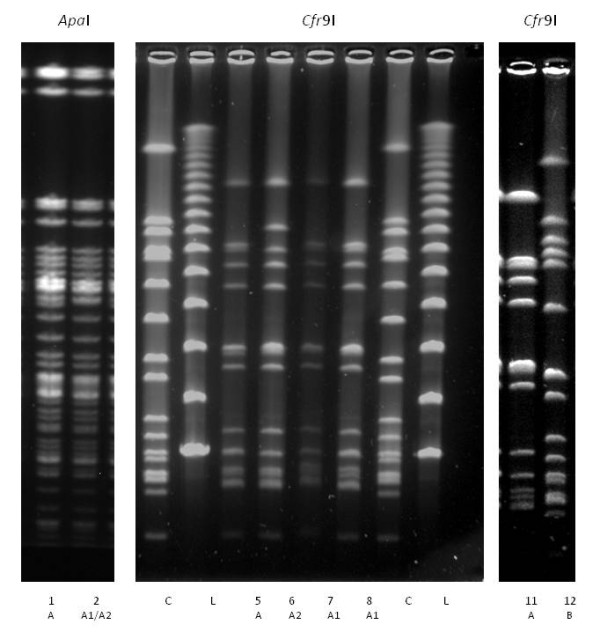
**PFGE pattern of representative MRSA isolates, *spa*-type t011 and t064**. Restrictions enzyme *Apa*I and *Cfr*9I. ***Apa*I: *Lane 1 ***- demonstrate the outbreak strain named pulsotype A. ***Lane 2 ***- pulsotype A1 and A2 isolates lacking one band. ***Cfr*9I**: ***Lane 5 ***- pulsotype A and ***lanes 7 + 8 ***- pulsotype A1, identical in *Cfr*9I gel. ***Lane 6 ***- pulsotype A2, one extra band. ***Lanes 11 ***and ***12 ***show difference between *spa-*type t011 (pulsotype A) and *spa-*type t064 (pulsotype B). ***C ***= control: *S. aureus *NCTC 8325 ***L ***= lambda ladder: Size range of 50-1,000 kb (PFG Marker, New England Biolabs Inc.) http://www.neb.com/nebecomm/products/productN0340.asp.

The PFGE pulsotypes are shown in Figure 2 and Table 1. Identical *Cfr*9I and *Apa*I band patterns were seen for nine of the 12 isolates, here named pulsotype A. According to the definition by Tenover et al. [47], those isolates were genetically indistinguishable and represented the same strain. One isolate, named pulsotype A1, lacked one band in the *Apa*I gel compared with pulsotype A, but was identical to pulsotype A in the *Cfr*9I gel. One isolate, named pulsotype A2, linked to Hospital 2 and found in 2009, about a year after the outbreak, showed A1 pattern in *Apa*I gel but also had an extra band in the *Cfr*9I gel. Pulsotypes A1 and A2 can be considered very closely related to the pulsotype A according to Tenover et al. [47], and any differences should be considered as normal genetic change over time. The third divergent isolate was of *spa-*type t064, showing a totally different gel pattern with both enzymes.

## Discussion

This study confirmed the first nosocomial outbreak of MRSA in a Swedish equine hospital. This was also the first MRSA infections notified in horses in Sweden, although one nasal carrier of *spa*-type t011 had been found in a screening survey of horses about six month earlier, in December 2007 [38]. As the screening was anonymous, it could not be determined whether there were connections between that finding and the subsequent outbreak. The outbreak strain belonged to the MRSA variant ST398, *spa*-type t011 [38,39]. This variant has been associated with livestock in Europe [12,19] and with horses [15,17,18,28,29], but had only been found in horses and a few humans [48] in Sweden.

The ST398, *spa*-type t011, isolates from Swedish horses were indistinguishable or very closely related according to PFGE, which corroborated the close relationship between them. The use of two restriction enzymes offered good discrimination between the isolates and revealed minor genetic change over time, from pulsotype A into pulsotype A1 in Hospital 1 (2010, Horse IX) and pulsotype A2 in Hospital 2 (2009, Horse VII). The pulsotypes A1 and A2 were isolated more than a year after pulsotype A and genetic events in a strain leading to changes in restriction site are not surprising, according to Tenover et al. [47] and indicated one circulating strain in the region.

It was not possible to find a plausible source of the outbreak since the index case could not be defined, as the first three horses infected had indirect cross-contacts within the hospital. Moreover, horses were not screened for MRSA on arrival at the equine hospital, probably because MRSA had not yet been identified as a risk in Sweden. Horses regularly travel to competitions and for breeding and change owners over regional and national borders. The MRSA in this study could well have originated from connections to livestock or horses in countries with a higher MRSA prevalence than Sweden. As MRSA had not been found in livestock in Sweden at that time, it seemed less likely that Swedish livestock would be the source [39,40]. The infection might even have derived from human carriers, such as people handling the horses, as professionals working with animals that carry MRSA ST398 have been reported to be colonised with this type [2,15,20-23]. The infected horse found during telephone tracing of the owners of horses that had undergone surgery during the outbreak period in Hospital 1 might have been overlooked otherwise, as the owner expressed no worry about secretions from the wound. This indicates a need for post-operative monitoring of horses in order to track down surgical complications, but also to communicate general information about signs of resistant bacteria to veterinary staff, horse owners and other people working with horses. The findings also point out a possible bias in estimated numbers of hospital-acquired infections based upon infections diagnosed solely within the hospital or clinic.

A different *spa*-type, t064, was introduced to Sweden almost two years after the outbreak in Hospital 2 (Table 1 Horse X). This *spa*-type has been reported in horses in other countries [12,15,49,50]. In Hospital 2 another horse (Table 1 Horse VIII) suffered from MRSA pulsotype A2, which was a minor genetic change of pulsotype A from the Hospital 1 outbreak. The two cases in Hospital 2 occurred approximately six months apart and was apparently not an outbreak, but the incident initiated a need for revision of the infection control procedures within the hospital. Such revision and improvement had already started at Hospital 1.

The majority of the MRSA infections identified in this study were SSI, confirming previous reports, although other infections do occur [8-10,12,14,15,17,28,51].

The zoonotic potential of MRSA means that infected and carrier horses could pose a risk to hospital staff and horse owners. Seven human cases of *spa*-type t011 were notified between 2006 and 2010 in Sweden [48], and the findings of *spa*-type t011 in people could be the result of animal to human transmission. It would be interesting to compare Swedish *spa*-type t011 MRSA isolates from people and horses by PFGE. In a Finnish study, isolates of *spa*-type t011 from people without horse contact showed a different PFGE pattern than t011 isolates from a veterinarian and horses in contact during a MRSA outbreak in an equine hospital [52].

The isolates tested here were resistant to all antimicrobials approved for equine use in Sweden and the only treatment options were drugs with a risk of inducing colitis in horses, e.g. erythromycin [53] and one for local use, fusidic acid. Spontaneous clearing of the infections without antimicrobial treatment was an encouraging observation, as there was no approved drug available in the studied cases and antimicrobial resistance is expanding and antimicrobials are considered a limited resource [54,55]. Wound care without antimicrobial treatment is a topic that needs to be further explored within veterinary practices, as well as in research.

The great variation in time from surgery to MRSA detection (15-52 days) was not acceptable, as delayed detection might influence the numbers of horses infected in an outbreak, as well as posing a hazard to staff. Some of the delay could be explained by negative cultures on the first sampling occasion and at least one unaware owner, the telephoned-traced case. Other reasons for delay were empirical antimicrobial treatment before sampling (Horse X) and delayed sampling (Horse I). Lack of awareness or neglect by veterinarians and others are hypothetical reasons for delays. The findings showed that infections should be taken seriously and would benefit from qualified sampling before antimicrobial use, although negative culture is a risk, as demonstrated in two of the cases in this study. The time between bacteriological sampling and laboratory confirmation of MRSA was also long in some cases. Culture for MRSA is a balance between speed of result, sensitivity, specificity and cost. A suitable sampling technique is vital for faster culture. Pre-washing of the top layer of an infected wound with sterile saline, water or likewise to remove decomposition flora might help to decrease the delay that arises from analysing mixed flora, especially if MRSA selective agar plates are not used and the growth of decomposition flora requires recultivation. However, there could also be delays because of lack of awareness or dismissal of MRSA by laboratories, since MRSA had not been detected previously in Sweden. Accurate diagnosis is important for all concerned; patient, owner, veterinary staff, etc. Verification of MRSA isolates by PCR is essential, since a false diagnosis of MRSA is as devastating as a true positive. Faster methods would be preferable to obtain a quick yes or no answer, but culture will still be required to get isolates for antimicrobial susceptibility testing and genetic and epidemiology studies. Real-time PCR as used for humans has been evaluated for detecting MRSA in horses, but proved unsatisfactory [56].

The MRSA outbreak raised an alarm for equine veterinarians and hospital staff in Sweden and increased their awareness about the need for improved infection control for their own safety and that of the horses. In addition, during the outbreak, the topic was reported and discussed frequently and thoroughly in the media, making the general public aware of the presence of MRSA bacteria in the equine population.

## Conclusions

MRSA was shown to be present in the Swedish equine population, although it appeared only regionally. The restriction enzymes *Cfr*9I and *Apa*I worked well for MRSA ST398 and demonstrated close genetic relatedness between most isolates from the horses tested, representing one strain. This and the correlation in time and space between the cases verified the occurrence of an outbreak. Later sporadic infections with the same strain indicated one circulating strain, but the primary source of the outbreak was never identified.

The outbreak highlights the importance of implementing preventive measures, such as sampling of infections, improved infection control with strict hygiene routines and antimicrobial strategies, to limit the spread of MRSA in animal hospitals. An encouraging observation was that infections cleared although no antimicrobial treatment was given. Wound care without antimicrobials would benefit from further research, as antimicrobials have become a limited resource within both human and veterinary medicine.

## Competing interests

The authors declare that they have no competing interests.

## Authors' contributions

KB initiated and designed the study together with UGA. KB interpreted the results and drew conclusions in discussion with the other authors. AL and AA set up the PFGE analyses and helped with the writing of that part. CJ was the contact at the hospital and provided information and records on the horses. All authors have read and approved the final manuscript.
